# fMRI evidence for the interaction between orthography and phonology in reading Chinese compound words

**DOI:** 10.3389/fnhum.2013.00753

**Published:** 2013-11-21

**Authors:** Jiayu Zhan, Hongbo Yu, Xiaolin Zhou

**Affiliations:** ^1^Department of Psychology, Peking UniversityBeijing, China; ^2^Center for Brain and Cognitive Sciences, Peking UniversityBeijing, China; ^3^Key Laboratory of Machine Perception (Ministry of Education), Peking UniversityBeijing, China; ^4^Key Laboratory of Computational Linguistics (Ministry of Education), Peking UniversityBeijing, China; ^5^ PKU-IDG/McGovern Institute for Brain Research, Peking UniversityBeijing, China

**Keywords:** compound word, pseudohomophone, reading, lexical processing, Chinese, fMRI

## Abstract

Compound words make up a major part of modern Chinese vocabulary. Behavioral studies have demonstrated that access to lexical semantics of compound words is driven by the interaction between orthographic and phonological information. However, little is known about the neural underpinnings of compound word processing. In this functional magnetic resonance imaging study, we asked participants to perform lexical decisions to pseudohomophones, which were constructed by replacing one or both constituents of two-character compound words with orthographically dissimilar homophonic characters. Mixed pseudohomophones, which shared the first constituent with the base words, were more difficult to reject than non-pseudohomophone non-words. This effect was accompanied by the increased activation of bilateral inferior frontal gyrus (IFG), left inferior parietal lobule (IPL), and left angular gyrus. The pure pseudohomophones, which shared no constituent with their base words, were rejected as quickly as non-word controls and did not elicit any significant neural activation. The effective connectivity of a phonological pathway from left IPL to left IFG was enhanced for the mixed pseudohomophones but not for pure pseudohomophones. These findings demonstrated that phonological activation alone, as in the case of the pure pseudohomophones, is not sufficient to drive access to lexical representations of compound words, and that orthographic information interacts with phonology, playing a gating role in the recognition of Chinese compound words.

## INTRODUCTION

Access to lexical semantics is a fundamental process in reading. It has been widely accepted that word meaning can be accessed in two ways. One way is through direct visual access, where visual features in the input are projected onto underlying orthographic representations in the lexicon, which are subsequently transformed directly into the activation of semantic properties. The other way is through a phonologically mediated process, wherein the orthographic input first activates lexical phonological representations and then activates semantic representations.

Psycholinguists disagree upon which route plays the predominant role in visual word recognition and to what extent the two pathways might be independent from each other (Coltheart, 1978; Van Orden, 1987; Seidenberg and McClelland, 1989; Van Orden et al., 1990; Coltheart et al., 1993; Plaut et al., 1996). Although it is widely accepted that phonological mediation plays a predominant role in accessing lexical semantics in reading alphabetic scripts (Frost, 1998), answers to these questions are more divergent for the Chinese logographic writing system. Unlike the alphabetical system, the basic meaningful units in the logographic system are characters, each of which corresponds to one morpheme and one syllable. However, given the limited number of syllables in the language, many morphemes or characters are homophonic, and these characters may or may not share orthographic features. Thus, a character’s pronunciation (i.e., syllabic representation) cannot be used to uniquely identify the meaning of the corresponding morpheme, reducing the efficiency of computation from orthographic input to semantic representation via phonological mediation.

Two contrasting views have been proposed for how lexical semantics is accessed in reading Chinese characters. One view, the “universal phonological principle,” is based on the idea that the phonological mediation plays the same predominant role in reading Chinese as it does with alphabetic scripts (Perfetti et al., 1992). However, the empirical findings in support of this view have proven difficult to replicate (see, for example, Chen and Shu, 2001; Xie and Zhou, 2003). The alternative view postulates that access to lexical semantics in Chinese is constrained by both phonology and orthography operating in interaction with each other, and that phonology has no inherently privileged role over orthography in driving semantic activation (Zhou and Marslen-Wilson, 1999, 2000).

Questions regarding the pathways to lexical semantics can also be asked for compound words, which consist of two or more constituent characters (morphemes) and which make up more than seventy percent of modern Chinese vocabulary (Institute of Language Teaching and Research, 1986). Taking advantage of the pseudohomophone effect in lexical decision, Zhou and Marslen-Wilson (2009) demonstrated that lexical access in reading Chinese compound words cannot rely solely on the combinatorial phonological information of the constituent characters, and that orthographic information plays an indispensable role in accessing the semantics of compounds. Pseudohomophones are non-words (e.g., *brane*) that sound like real words but are written differently. It has consistently been observed that when making lexical decisions, participants need more time to reject the pseudohomophones than the match non-pseudohomophone non-words (e.g., *brune*). This effect has been taken as evidence that the visual input of a pseudohomophone activates the corresponding phonological representation (e.g., /brein/), and this activation automatically spreads to all semantic representations corresponding to this phonological representation, including the one corresponding to the base word of the pseudohomophone (e.g., *brain*). The semantic activation of the base word interferes with the processing system by slowing down the “no” decision.

Zhou and Marslen-Wilson (2009) created pseudohomophones in Chinese by replacing one (e.g., 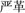 yan[2]ge[2]) or both (e.g., 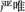 yan[2]ge[2]) constituents of two-character compound words (e.g., 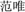 (yan[2]ge[2], *strict*; the number in brackets indicating the tone of the syllable). They found that mixed pseudohomophones sharing either the first or second constituent with their base words were more difficult to reject than control non-words, but pure pseudohomophones sharing no constituents with their base words did not show this effect. Moreover, the pseudohomophone effect in lexical decision interacts with the frequency of the shared constituents of mixed pseudohomophones: while pseudohomophones based on high frequency words with high or low frequency characters and pseudohomophones based on low frequency words with high frequency characters were more difficult to reject than control non-words, pseudohomophones based on low frequency words and with low frequency characters did not show a significant effect. The authors interpreted the pseudohomophone effect as reflecting the semantic activation of base words by both the orthographic and phonological information conveyed in mixed pseudohomophones. Taking together other findings, Zhou and Marslen-Wilson (2009) argued that the direct mapping from orthography to semantics plays a dominant role in Chinese compound word recognition and this pathway acts in an interactive manner with phonological information in driving semantic activation.

The main purpose of the current study was to examine the neural basis of the pseudohomophone effect and to provide further evidence for the interaction between orthography and phonology in reading Chinese compound words. To this end, we asked participants to carry out lexical decisions to mixed and pure pseudohomophones as in Zhou and Marslen-Wilson (2009) and measured their brain activity with functional magnetic resonance imaging (fMRI). Specifically, we attempted to find the brain regions underlying the pseudohomophone effects and to see whether or not these regions would show a pattern of activation parallel to the pattern of the behavioral finding, i.e., a significant pseudohomophone effect for mixed pseudohomophones and an absence of this effect for pure pseudohomophones. We will also relate our findings to those on the processing of single-character words and to those on alphabetic pseudohomophones.

Although there has been no neuroimaging study on how the processing of Chinese compound words is influenced by the processing of their constituent characters, previous research on Chinese characters may provide some clues as to where we might expect to find activations for different processes involved in the recognition of compound words. Studies explicitly asking participants to carry out phonological tasks, such as rhyme judgment (Xue et al., 2005), homophone judgment (Kuo et al., 2004; Dong et al., 2005), and naming (Kuo et al., 2003; Lee et al., 2004; Liu et al., 2006), have consistently implicated left inferior parietal lobule (IPL) and the dorsal part of the left inferior frontal gyrus (IFG). The left IPL is sensitive to the conflict between orthographic and phonological information, as in naming inconsistent characters (i.e., characters that share the same phonetic radical but are pronounced differently; Lee et al., 2004). Since the resolution of such conflicts relies on the extraction of the relationship between orthographic and phonological forms of the characters, it has been suggested that this region is associated with transformation and integration between orthography and phonology (Booth et al., 2002, 2006; Newman and Joanisse, 2011). Given that phonological information is automatically activated by orthographic input (Zhou and Marslen-Wilson, 2000) and that this activation may interact with orthographic information to constraint access to lexical semantics (Zhou and Marslen-Wilson, 1999, 2009), we expected to observe IPL activation for mixed pseudohomophones, relative to their non-word controls, but not for pure pseudohomophones.

Previous neuroimaging studies have also examined neural correlates of semantic processes in character processing by using tasks such as semantic relatedness judgment (Tan et al., 2001; Dong et al., 2005; Xue et al., 2005). These studies showed the activation of the left angular gyrus and the left IFG. In a recent study, Chou et al. (2009) manipulated the strength of semantic association between two characters and found that the stronger semantic association elicited greater activation in the left angular gyrus and that the weaker semantic association elicited greater activation in the left IFG during semantic relatedness judgment. The authors suggested that activation of the angular gyrus is driven by the overlapping semantic features shared by the character pairs, whereas activation of the left IFG reflects the effortful retrieval and selection of appropriate semantic features. Given that lexical decision to Chinese compound words and non-words is mainly based on semantic activation (Zhou et al., 1999; Zhou and Marslen-Wilson, 2009), and given that IFG is involved in a number of different processes in language comprehension, we expected to observe the activation of both the left angular gyrus and the left IFG only for mixed pseudohomophones, relative to control non-words, and not for pure pseudohomophones (assuming that the pure pseudohomophones are not sufficient to activate the semantic representation of the base words).

## MATERIALS AND METHODS

### PARTICIPANTS

Nineteen undergraduate and graduate students (10 males, mean age 22) participated in our experiment. They were native speakers of Chinese and were right-handed as assessed by the Chinese Handedness Questionnaire (Li, 1983). They had normal or corrected-to-normal vision, and none of them reported to have a history of neurological or psychiatric disorder. Informed written consents were obtained from all the participants prior to scanning. This study was approved by the Ethics Committee of the Department of Psychology at Peking University. In the final data analysis, three participants (one male and two females) were excluded, one for chance-level accuracy in lexical decision and two for excessive head movements.

### STIMULI AND PROCEDURES

A total of 120 two-character compound words were chosen as base words. All of these words, like most compound words in Chinese, were phonologically unambiguous. Phonological ambiguity means that no other compound words have the same phonological forms as the base words used in this study. The mean frequency of the base words was 151 per million. The average character frequencies were 756 per million for the first constituent character and 615 per million for the second constituent character.

Two types of pseudohomophones were created according to whether the second constituents (“mixed”) or both constituents (“pure”) of the base words were replaced with orthographically dissimilar homophonic characters. We did not include pseudohomophones that were created by replacing the first characters of the base words, because this type of pseudohomophones showed the same pattern of effects as the mixed pseudohomophones used here (Zhou and Marslen-Wilson, 2009). Control non-words were created by recombining the first and second constituents of pseudohomophones. In other words, the pseudohomophones and the corresponding control non-words used the same set of characters, although the mixed and the pure pseudohomophones differed in their initial characters. Examples of pseudohomophones and their controls derived from the base words are presented in **Table 1**. Properties of the constituent morphemes of pseudohomophones (and the corresponding control non-words) are summarized in **Table 2**. These properties included the average character frequency (per million), visual complexity (in terms of the number of strokes per character), and the average “productivity,” which indexed the number of compound words that contained the characters as constituents.

**Table 1:** Experimental design.

	Pseudo		Control	
Mixed	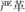	yan[2]ge[2]	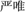	yan[2]wei[2]
	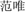	fan[4]wei[2]	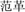	fan[4]ge[2]
Pure	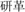	yan[2]ge[2]	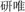	yan[2]wei[2]
	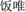	fan[4]wei[2]	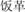	fan[4]ge[2]

*Note: Pseudohomophones in the table are derived from the base words* 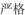 *(yan[2]ge[2], strict) and* 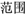 *(fan[4]wei[2], scope). The first characters in the “mixed” group are also the first characters of base words*.

**Table 2:** Properties of stimuli.

	First character			Second character		
	Number of strokes	Character frequency	Total productivity	Number of strokes	Character frequency	Total productivity
Mixed	8.4	756	54.9	8.1	648	37.7
Pure	8.8	784	36.7	8.1	648	37.7

*Number of strokes measures visual complexity of the character; Total productivity refers to the number of words contain this character as a constituent.*

The critical stimuli were assigned into four test versions using a Latin square design. Each version was composed of 60 pseudohomophones and 60 control non-words; half of each type were from “Mixed” group and the other half were from “pure” group. Pseudohomophones and control non-words created from the same base words were split into different versions. Each version additionally had 120 filler words that were the same across the four versions. Each participant received one version in which pseudohomophones, control non-words, and word fillers were presented in a pseudo-random order (with the restriction that no more than three consecutive trials were from the same category). In each trial, participants were asked to decide as quickly and as accurately as possible, by pressing the “yes” or “no” button, whether the two characters presented on the screen formed a real word or not. For half the participants, the “yes” button was pressed by the right thumb and the “no” button by the left thumb; for the other half, the mappings between fingers and buttons were reversed.

For each trial, an eye fixation sign (“+”) was first presented at the center of the screen for 250 ms, followed by a 100 ms blank interval; the fixation was then presented again for another 250 ms, creating a flick that could more firmly capture attention. A word or non-word, subtending a visual angle of about 2.5° horizontally and 1.25° vertically, was finally presented for 400 ms for lexical decision. The interval between the disappearance of the last stimuli and the appearance of the next fixation sign was randomized between 4000 and 6000 ms to improve the ability to detect regions of BOLD signal changes (Serences, 2004).

The 240 trials were scanned in one session, lasting about 25 min. A fixation sign was displayed at the beginning of the session for 10 s to allow the scanner to reach stability. Before entering the scanner, all the participants completed a practice session consisting of 24 stimuli with similar compositions of stimuli as the formal test.

### fMRI DATA ACQUISITION AND ANALYSIS

Functional images were acquired on a 3-T Siemens Trio system at the Institute of Biophysics, Chinese Academy of Sciences, using a T2^*^-weighted echo planar imaging (EPI) sequence, with 2 s repetition time, 30 ms echo time, and 90° flip angle. Each image consisted of 32 axial slices covering the whole brain. Slice thickness was 3 mm and inter-slice gap was 0.75 mm, with a 220 mm field of view, 64 × 64 matrix, and 3 mm × 3 mm × 3 mm voxel size.

Data were pre-processed with Statistical Parametric Mapping (SPM) software SPM8 (Welcome Department of Imaging Neuroscience, London, ). The first five volumes were discarded to allow stabilization of magnetization. Images were realigned to the sixth volume for head movement. Participants whose head movements did not exceed 3 mm were included in the final data analysis. A temporal high-pass filter with a cutoff frequency of 1/128 Hz was used to remove low-frequency drifts in an fMRI time series, and smoothed with a Gaussian kernel of 8 mm full-width half-maximum (FWHM).

Statistical analysis was based on the general linear model (GLM). The hemodynamic response to each event was modeled with a canonical hemodynamic response function (HRF) with its temporal derivative. We define seven regressors: four corresponded to the correctly judged trials in the four conditions (interested regressors), one corresponded to the correctly judged trials for filler words, one corresponded to the incorrectly judged trials and outlier, and one corresponded to the button press. The six rigid body parameters were also included to correct for the head motion artifact. The onset of the critical regressors was set to the appearance of the pairs of characters. We rendered the SPMs at an uncorrected voxel threshold of *p* < 0.001 and report maxima with a cluster size of *p* < 0.05 corrected for multiple comparisons and adjusted for the entire brain, unless otherwise stated. We conducted spatially restricted region of interest (ROI) analysis using anatomically defined ROI masks based on the automatic anatomical labeling (AAL) system (Maldjian et al., 2003) with voxel threshold *p* < 0.05 (FWE-corrected) and cluster size threshold of 20 voxels.

Effective connectivity analysis was performed using the Dynamic Causal Modeling tool in SPM. Bilinear DCM, which was used in this study, is featured by three different sets of parameters (Friston et al., 2003): (1) the “intrinsic” connectivity representing the latent connectivity between brain regions in the absence of experimental perturbations; (2) the “modulatory” connectivity representing the changes imposed on the intrinsic connectivity by experimental perturbations; and (3) the “input” representing the driving influence on brain regions by external perturbations. Since we were interested in seeing whether the semantic representation can be accessed through a phonologically mediated route (as the strong phonological view argues) or through interaction between orthographic and phonological information, the model was restricted to the phonological and semantic related regions activated for the main effect of pseudohomophones (i.e., IPL, MNI coordinates: -46, -46, 44; IFG, MNI coordinates: -46, 8, 22; see Results). Specifically, we examined whether the activity of this network was modulated by the orthographic information carried by the pseudohomophones (i.e., the type of pseudohomophones). For each volume of interest (VOI), a time series was extracted as the first principal component of all voxel time series within a sphere (radius 4 mm) centered on the group maximum. We constructed and compared four models, which had the same input region (i.e., the left IPL) and intrinsic connectivity pattern (bidirectional connectivity between IFG and IPL) but differed in the way in which the experimental manipulations (i.e., mixed vs. pure pseudohomophone conditions) modulated the connectivity. We chose the left IPL as the input region since it is implicated in orthography-to-phonology mapping. For Model 1 and Model 2, the modulatory effects were exerted on the IPL-to-IFG intrinsic connectivity, with only the mixed (Model 1) or both mixed and pure pseudohomophones (Model 2) as the modulatory factors. For Model 3 and Model 4, the modulatory effects were exerted on the IFG-to-IPL intrinsic connectivity, with only the mixed (Model 3) or both mixed and pure pseudohomophones (Model 4) as the modulatory factors. The four models were compared using random-effect Bayesian Model Selection (BMS; Penny et al., 2004; Stephan et al., 2009), by which the “exceedance probability” (the probability of each model being more likely than any other model) of each model was calculated. Effective connectivity strength was estimated based on the model with the highest exceedance probability (i.e., the winning model).

## RESULTS

### BEHAVIORAL RESULTS

**FIGURE 1:**
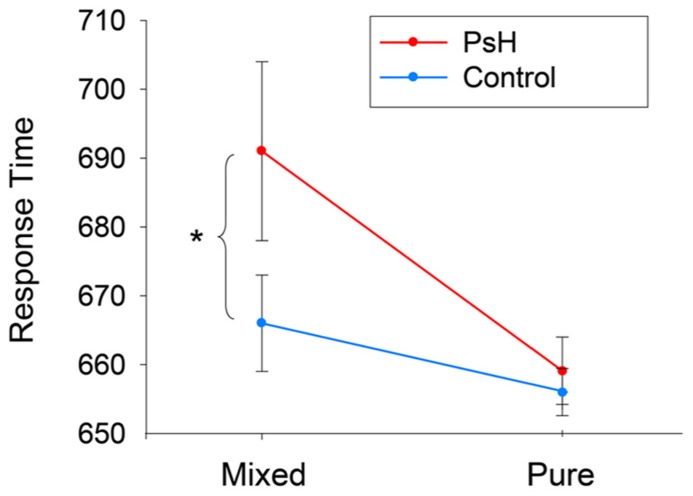
**Mean response times of lexical decision to two mixed and pure pseudohomophones and their respective controls.** ^*^p < 0.05.

**Figure 1** shows the mean response times (RTs) for the final 16 participants (4 for each version of stimuli) based on correct, untrimmed responses. Repeated-measures ANOVAs were conducted separately for RTs and error rates, with both stimulus type (pseudohomophone vs. control) and stimulus group (mixed vs. pure) as within-participant factors. For RTs, the main effect of stimulus type was not significant, although the main effect of stimulus group was, *F*(1,15) = 20.917, *p *< 0.001, with the stimulus RTs in the Mixed group significantly slower than the stimulus RTs in the Pure group. Importantly, the interaction between stimulus type and stimulus group was significant, *F*(1,15) = 3.96, *p* = 0.06, such that RTs for mixed pseudohomophones (mean = 692 ms, SD = 161 ms) were significantly longer than RTs for the controls (667 ± 167 ms), *t*(15) = 2.74, *p *< 0.05, whereas no difference was found between the pure pseudohomophones (658 ± 167 ms) and their controls (654 ± 176 ms), *t*(15) = 0.43, *p *> 0.1. For error rates, there was a significant main effect of stimulus type, *F*(1,15) = 8.94, *p *< 0.01, and a significant main effect of stimulus group, *F*(1,15) = 21.42, *p *< 0.001, indicating that, in general, participant responses were more error-prone to pseudohomophones than to control non-words and more error-prone to the Mixed group than to the pure group.

### fMRI RESULTS

#### General linear model analysis

**FIGURE 2:**
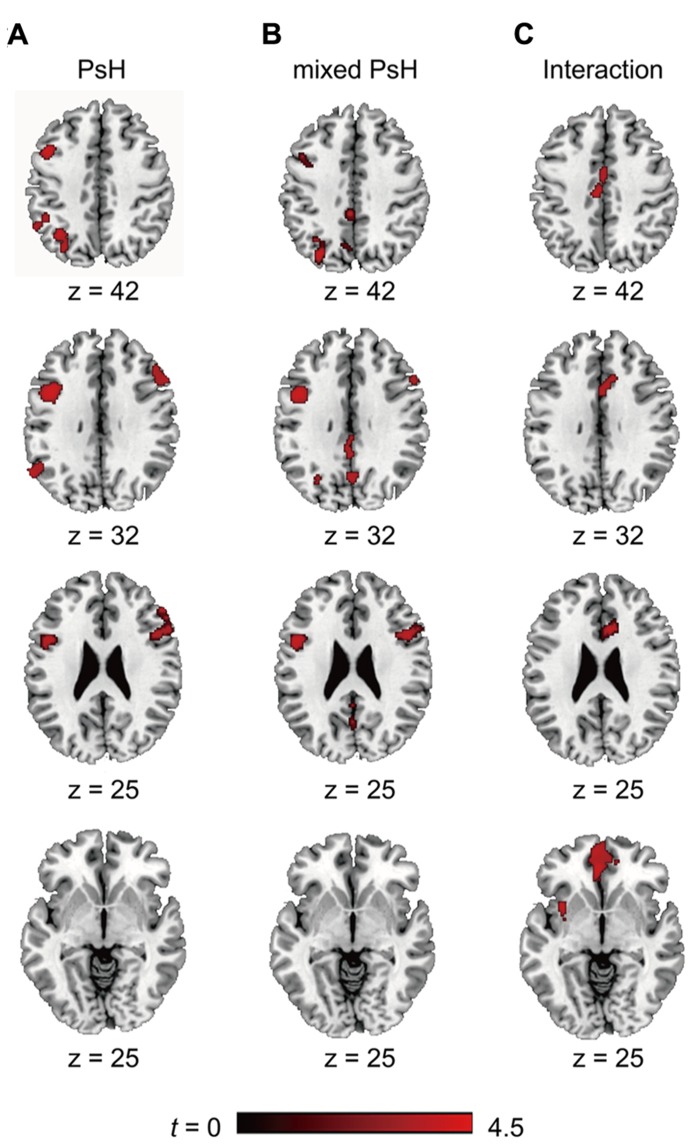
**The activated brain areas in different contrasts, PsH = pseudohomophone.** **(A)** Significant clusters as revealed by “PsH > Controls”; **(B)** Significant clusters as revealed by “mixed PsH > Controls”; **(C)** Significant clusters as revealed “(mixed PsH > Controls) > (pure PsH > Controls).”

We first identified the brain regions involved in the main effect of pseudohomophones (collapsed over the Mixed and Pure groups). Compared with control non-words, reading pseudohomophones invoked greater activity in the bilateral IFG (left BA44 and right BA 44/45/48), left IPL (BA40), and left angular gyrus (BA7; **Figure 2A**).

As we were interested in the differential activations associated with different types of pseudohomophones, we contrasted mixed and pure pseudohomophones with their controls respectively. Compared with controls, mixed pseudohomophones activated the bilateral IFG, left angular gyrus, as well as the left insular, middle and posterior cingulated cortex (**Figure 2B**). The activation of left IPL, however, failed to reach the statistical threshold in the whole-brain analysis after separating the two types of pseudohomophones. Since the left IPL has been consistently implicated in phonological processing and may play an important role in accessing lexical representations of the base words in this study, we therefore conducted a spatially restricted analysis of this region using anatomically defined ROI masks based on the AAL system. A significant cluster was found for the contrast “mixed pseudohomophones > controls” within the ROI (MNI: -48, -42, 44; *k* = 476). For pure pseudohomophones, no brain regions reached the cluster level threshold.

We also examined the interaction of stimulus type (pseudohomophone vs. non-word) by stimulus group (mixed vs. pure) by conducting the contrast between “mixed pseudohomophones > controls” and “pure pseudohomophones > controls.” This contrast revealed activations in the medial orbitofrontal cortex and anterior cingulate cortex (**Figure 2C**).

**FIGURE 3:**
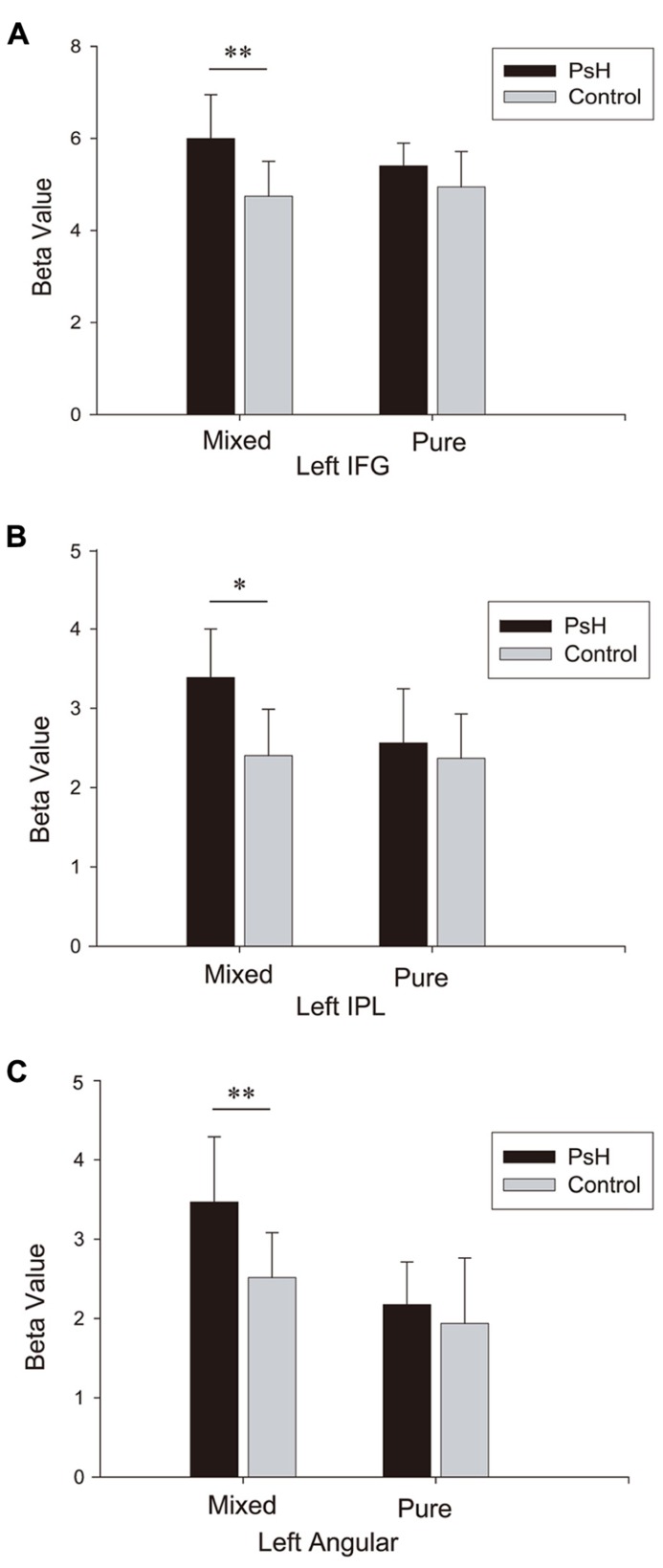
**Beta values for three regions of interest: left IFG (A), left IPL **(B)**, and left angular gyrus **(C)**, as revealed by the contrast “PsH > Controls**.”

To show more detailed information concerning the activity in the regions revealed in the main contrast (pseudohomophone vs. non-word), we computed the average beta values of these regions and conducted ROI analysis. Each ROI was defined as a cube with a side length of 5 mm, centered at the maximum coordinates of a cluster listed in **Table 3**. **Figure 3** plots the beta values in these ROIs. Statistical tests showed significant main effects of stimulus type for all the three regions, consistent with the main contrast: left IFG, *F*(1,15) = 8.31, *p *< 0.05; left IPL, *F*(1,15) = 8.75, *p *= 0.01; and left angular gyrus, *F*(1,15) = 17.42, *p *= 0.001. Importantly, the interaction between stimulus type and stimulus group was significant for the left IPL and IFG:* F*(1,15) = 4.33, *p *= 0.055 and* F*(1,15) = 5.16, *p *< 0.05, respectively. The same trend was also observed for the left angular gyrus* F*(1,15) = 3.57, *p *= 0.078. Tests of simple effects showed that activations were significantly higher for the mixed pseudohomophones than for the controls: *t*(15) = 3.09, *p *< 0.01 for IFG; *t*(15) = 3.83, *p *< 0.05 for IPL; and *t*(15) = 3.96, *p *= 0.01 for angular gyrus. However, no differences were found between the pure pseudohomophones and their controls:* t*(15) = 1.57, *p *= 0.14 for IFG; *t*(15) = 0.71,* p *= 0.49 for IPL; and *t*(15) = 1.02,* p *= 0.32 for left angular gyrus. Clearly, the pattern of effects in the ROI analysis here is consistent with the findings in the above comparisons for the two types of pseudohomophones.

**Table 3:** MNI coordinates of the activation foci revealed by three contrasts.

			PsH – control						Mixed PsH – control						Interaction					
						MNI coordinates						MNI coordinates						MNI coordinates		
Regions	H	BA	*P*_FWE_	Max *z*-value	Voxel	*x*	*y*	*z*	*P*_FWE_	Max *z*-value	Voxel	*x*	*y*	*z*	*P*_**FWE**_	Max *z*-value	Voxel	*x*	*y*	*z*
IFG	L	44	0.000	4.82	490	-46	8	22	0.001	4.55	349	-46	8	24		–	–	–	–	–
	R	45	0.005	3.84	276	50	14	28	0.053	3.82	152	50	14	26		–	–	–	–	–
Insular	L	48		–	–	–	–	–	0.040	4.19	167	-30	18	-10		–	–	–	–	–
mOFG	L			–	–	–	–	–		–	–	–	–	–	0.000	4.24	795	-2	40	-8
ACC	L			–	–	–	–	–		–	–	–	–	–		4.05	^a^	-6	36	-8
	R			–	–	–	–	–		–	–	–	–	–		4.04	380	12	20	28
MCC	L			–	–	–	–	–	0.041	3.99	166	-4	-38	38	0.001	4.11	^a^	-6	20	44
PCC	L/R			–	–	–	–	–		3.27	^a^	0	-32	30		–	–	–	–	–
IPL	L	40	0.026	3.86	189	-46	-46	44			–	–	–	–		–	–	–	–	–
Angular	L	7	0.021	3.93	199	-34	-58	42	0.020	3.18	223	-32	-60	42		–	–	–	–	–

*PsH, pseudohomophone; IFG, inferior frontal gyrus; mOFG, medial orbitofrontal gyrus; ACC, anterior cingulated cortex; MCC, middle cingulated cortex; PCC, posterior cingulated cortex; IPL, inferior parietal lobe.*

#### Effective connectivity analysis

**FIGURE 4:**
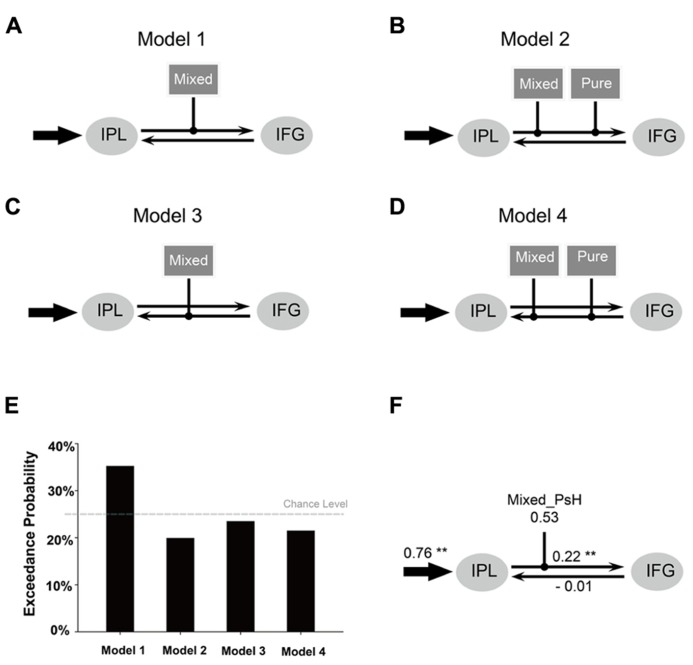
**Outline of the four DCM models tested in the present study.** **(A–D)** Results of Bayesian Model Selection **(E)**. The estimated DCM parameters of the winning model **(F)**. Arrows represent driving input into regions, or intrinsic connections between regions. Lines with black dots at their ends indicate modulations of the intrinsic connections by the task. Mixed = mixed pseudohomophone; Pure = pure pseudohomophone. ^*^^*^p < 0.05 (Bonferroni).

**Figures 4A–D** presents four DCM models for the connectivity between the left IPL and the left IFG. Result of BMS showed that Model 1 had an exceedance probability of 35.2%, which was greater than the exceedance probability of all the other models (**Figure 4E**). The estimated connectivity strength of Model 1 yielded the following results (**Table 4**; **Figure 4F**): the input to the left IPL by mixed pseudohomophones, but not pure pseudohomophones, was significantly greater than zero (*p* < 0.01, Bonferroni-corrected for multiple comparisons). The intrinsic connectivity from the left IPL to the left IFG, but not the other way around, was significantly larger than zero (*p* < 0.05, Bonferroni-corrected for multiple comparisons). Finally, the modulation of the intrinsic connectivity from the left IPL to the left IFG by the mixed pseudohomophones was positive and was significantly greater than zero (*p *< 0.05).

**Table 4:** Average parameter estimates of Model 1, their standard error, and their significances in one-sample *t*-tests.

	Mean	Standard error	*t*	*df*	*p*
Input of mixed PsH into left IPL	0.76	0.17	4.41	15	<0.01
Input of pure PsH into left IPL	0.22	0.19	1.17	15	0.26
Intrinsic connectivity left IPL ? left IFG	0.22	0.22	2.56	15	<0.05
Intrinsic connectivity left IFG ? left IPL	-0.01	-0.01	-0.08	15	0.94
Effect of mixed PsH on connectivity left IPL ? left IFG	0.53	0.22	2.36	15	<0.05

*PsH, pseudohomophone.*

## DISCUSSION

This study provides the first neural evidence for the processing of Chinese compound words. By applying a lexical decision task to pseudohomophones, we demonstrated that, compared with control non-words, only pseudohomophones that share one constituent with their corresponding base words were more difficult to reject, and that this effect was found in language-related brain regions such as the left IFG, left IPL, and left angular gyrus. Pure pseudohomophones that had no orthographic similarity to the base words were no more difficult to reject and had no obvious brain activation compared with non-word controls. These results suggest that an interaction between orthography and phonology, rather than a predominant phonological mediation, is responsible for the semantic activation in reading Chinese compound words. The connectivity analysis further showed that the link between the left IPL and IFG is not simple phonological, as mixed pseudohomophones, not pure pseudohomophones, enhanced the functional connectivity between these two brain areas.

The left IPL has been shown to be involved in phonological processing for both reading alphabetic scripts (e.g., Gold and Buckner, 2002; Gold et al., 2005) and reading Chinese (e.g., Booth et al., 2006; Liu et al., 2009). In the current study, the left IPL was activated only by the mixed, not the pure pseudohomophones, indicating that orthographic input gates the neural processing of phonological information associated with compound words. The left IPL serves to construct the phonological representation of Chinese compound words by integrating the phonological and orthographic information of its constituent morphemes. This is in line with a previous finding that the left IPL is involved in lexical decision to English orthographically similar homophones (*dear* vs. *deer*), as compared with non-homophone control words (Newman and Joanisse, 2011). Disambiguating these homophones also requires the integration of orthographic and phonological information.

The dorsal part of the left IFG (BA44) has been found to serve as a control center that collaborates with posterior brain regions for phonological retrieval or selection (e.g., Poldrack et al., 1999; Burton et al., 2000; Fiebach et al., 2002; Gold and Buckner, 2002; McDermott et al., 2003; Gold et al., 2005; Joseph et al., 2006; Liu et al., 2009). Moreover, activation of this part of the left IFG has also been observed in tasks that require access to the meaning of words (Thompson-Schill et al., 1997; Wagner et al., 2001; Gold and Buckner, 2002; Gold et al., 2005; Xue et al., 2005; Chou et al., 2009). Given that the pseudohomophone effect in lexical decision to Chinese compounds reflects the semantic activation of base words (Zhou and Marslen-Wilson, 2009) and given the absence of IFG activation for pure pseudohomophones, we argue that the activation of the left IFG in responding to mixed pseudohomophones reflects the process of retrieving semantic properties of base words. This semantic processing is interactively supported by the phonological representation of the compounds (i.e., the combination of syllabic representations) and appropriate orthographic input (the character shared between the base word and the mixed pseudohomophone).

One could argue that the activation of the left IFG might serve as a top-down modulation of the activation in the left IPL (Cao et al., 2008). However, our effective connectivity analysis found only intrinsic connectivity from the left IPL to left IFG, not the other way around, indicating a unidirectional impact of IPL activation on IFG activation. The connectivity from the left IPL to left IFG has also been found in previous studies on word recognition (Levy et al., 2008, 2009) and rhyme judgment (Cao et al., 2008), indicating its role in phonological analysis. However, the fact that this connectivity was only enhanced by the mixed pseudohomophones, and not by the pure pseudohomophones, suggests that orthographic information plays a vital role in this “phonology-to-semantic network,” at least for the processing of compound words. In other words, using phonological information to access lexical semantics relies on appropriate orthographic support. Thus, in reading Chinese compound words, orthographic and phonological information is integrated in the left IPL, and this integration is then projected to the left IFG for the retrieval of lexical semantics.

Previous neuroimaging studies demonstrated the involvement of the left angular gyrus in processing anomalous words embedded in sentences (Ni et al., 2000; Friederici et al., 2003; Newman et al., 2003), suggesting that it functions to integrate individual concepts into larger ones (Lau et al., 2008; Binder et al., 2009). Indeed, Binder et al. (2009) suggested that this region also functions as a communication hub where different types of intra-lexical information, such as orthography, phonology, and semantics, converge and interact. Zhou et al. (1999) suggested that, in the real-time processing of a Chinese compound word, both semantic representation of the whole word and the semantic representations of its constituent morphemes are activated in parallel, and that the semantic activation of constituent morphemes can be consistent or in conflict with the activation of the whole word. It is plausible that the activation of the left angular gyrus for mixed pseudohomophones may reflect this parallel activation and integration. Further studies are needed to investigate systematically the neural basis of competition and collaboration between semantic activation of whole words and constituent morphemes.

Direct contrast between the pseudohomophone effects for the mixed and pure pseudohomophones (i.e., the interaction analysis) did not show any activation of language-related areas such as the left IPL, IFG, and angular gyrus, although the interaction* was* found in the ROI analysis for these regions. Instead, the whole-brain interaction analysis showed the activation of the anterior cingulate cortex and medial orbitofrontal gyrus. These regions have long been associated with conflict detection and cognitive control (van Veen et al., 2001; Milham et al., 2003; Stephan et al., 2003; Ye and Zhou, 2009). It is possible that a more effortful control process is needed when making “no” responses to mixed pseudohomophones when the corresponding base words are activated not only by the phonological and orthographic information associated with the pseudohomophones but also by the morphemic representation for the shared (the first constituent) morphemes (Zhou et al., 1999; Zhou and Marslen-Wilson, 2009). The activation of the right IFG for mixed pseudohomophones also reflects the involvement of cognitive control in processing the mixed pseudohomophones (Xue et al., 2008; Vigneau et al., 2011).

We may need to rule out an alternative account for the brain activations for mixed pseudohomophones. This account states that the difficulty in rejecting mixed pseudohomophones and the associated brain activations reflect the processing of orthographic information for the characters shared between the pseudohomophones and the base words; phonological information and its interaction with orthographic information plays no role in processing the compound words. Although our experimental design does not allow us to rule out this account completely, the pattern of brain activations here and our unpublished behavioral data suggest that the pseudohomophone effect observed for mixed pseudohomophones was not driven purely by orthographic information. Compound non-words that were orthographically similar to the base words but shared no morpheme with the base words were only slightly more difficult to reject than non-words composed of randomly chosen characters. A large number of studies have also demonstrated that the processing of orthographic information alone occurs mainly in the occipitotemporal cortex (e.g., Kuo et al., 2004; Liu et al., 2008; Chan et al., 2009; Wang et al., 2011), whereas here we observed activations in parietal and frontal regions. Indeed, the orthographic properties of the pseudohomophones and control non-words were perfectly matched in this study, as the pseudohomophones and the corresponding non-word controls used the same sets of characters.

How do we relate the current findings with those for single-character words? As we reviewed previously, single-character words activate the left IPL in a variety of tasks that require explicit phonological processing (e.g., Booth et al., 2006; Liu et al., 2009). This phonological processing may take the form of linking directly the orthographic information with syllabic representation. For the compound words, however, in additional to activate syllabic representations for constituent morphemes, the co-occurrence information for the two constituent morphemes should also be activated (Zhou et al., 1999; Zhou and Marslen-Wilson, 2009). This activation of co-occurrence information at the left IPL, which plays a part in constructing phonological representations for the whole word by integrating constituents’ representations (i.e., syllables), must be supported by appropriate orthographic information, as only the mixed pseudohomophones, not the pure pseudohomophones, showed the IPL activation. Given that the activation of this co-occurrence information is modulated by the frequency of the whole words and the constituent morphemes (Zhou and Marslen-Wilson, 2009), an interesting issue for further studies is how the activation in the left IPL is affected by these factors.

In previous research, the left angular gyrus did not show activation for single-character words in tasks that might or might not activate semantic information in the previous research (e.g., Kuo et al., 2003; Lee et al., 2004; Liu et al., 2006), but it did show up in a task judging the semantic relatedness between two characters (Chou et al., 2009). Here, for mixed pseudohomophones, we also observed the left angular gyrus activation. It is possible that this region plays a general role in semantic integration, as the processing of compound words may need to evaluate and integrate the semantic properties of constituent morphemes and whole words (Zhou et al., 1999).

Finally, how do we relate the current findings with those for alphabetic pseudohomophones? Compared with the control non-words sharing most letters with the pseudohomophones, English pseudohomophones activated the left IFG, precentral gyrus, and cingulate cortex in a lexical decision task (Newman and Joanisse, 2011, see also Edwards et al., 2005). The authors attribute the pseudohomophone effects observed in these regions to phonologically mediated activation of the base words. However, these authors did not make explicit that their pseudohomophones shared most orthographic information with the base words (i.e., these pseudohomophones were more similar to the mixed pseudohomophones rather than pure pseudohomophones in this study). In this study, we also observed the left IFG activation for the mixed pseudohomophones. Importantly, we additionally observed the left IPL and angular gyrus activation. As we argued earlier, this additional activation, plus the evident connectivity between the IPL and IFG, may indicate that the role of orthographic information in the “phonologically mediated” semantic activation. The processing of orthographic information plays an indispensable role in reading logographic Chinese and accessing lexical semantics (Zhou and Marslen-Wilson, 1999, 2000, 2009; Zhou et al., 1999).

## CONCLUSION

By asking participants to carry out lexical decision to Chinese compound words and by introducing different types of pseudohomophones, we found a significant delay in rejecting mixed pseudohomophones and no such effect for pure pseudohomophones. Neurally, relative to non-word controls, mixed pseudohomophones activated the bilateral IFG, left IPL, left angular gyrus, and regions related to cognitive control; the processing of mixed pseudohomophones modulated the “phonological pathway” from the left IPL to the left IFG. For pure pseudohomophones, they showed no significant brain activation as compared with their non-word controls. These findings provide support for an interactive view according to which access to lexical semantics in reading logographic Chinese is driven by the interaction between orthographic and phonological information.

## Conflict of Interest Statement

The authors declare that the research was conducted in the absence of any commercial or financial relationships that could be construed as a potential conflict of interest.
